# Patient-derived bladder cancer xenografts in the preclinical development of novel targeted therapies

**DOI:** 10.18632/oncotarget.3974

**Published:** 2015-05-27

**Authors:** Wolfgang Jäger, Hui Xue, Tetsutaro Hayashi, Claudia Janssen, Shannon Awrey, Alexander W. Wyatt, Shawn Anderson, Igor Moskalev, Anne Haegert, Mohammed Alshalalfa, Nicholas Erho, Elai Davicioni, Ladan Fazli, Estelle Li, Colin Collins, Yuzhuo Wang, Peter C. Black

**Affiliations:** ^1^ The Vancouver Prostate Centre and Department of Urologic Sciences, University of British Columbia, Vancouver, BC, Canada; ^2^ Department of Urology, Johannes Gutenberg University, Mainz, Germany; ^3^ Department of Cancer Endocrinology, BC Cancer Agency, Vancouver, BC, Canada; ^4^ Research and Development, GenomeDx Biosciences, Vancouver, BC, Canada

**Keywords:** bladder cancer, muscle invasive bladder cancer, targeted therapy, patient-derived cancer xenografts, animal model

## Abstract

Optimal animal models of muscle invasive bladder cancer (MIBC) are necessary to overcome the current lack of novel targeted therapies for this malignancy. Here we report on the establishment and characterization of patient-derived primary xenografts (PDX). Patient tumors were grafted under the renal capsule of mice and subsequently transplanted over multiple generations. Patient tumor and PDX were processed for analysis of copy number variations by aCGH, gene expression by microarray, and expression of target pathways by immunohistochemistry (IHC). One PDX harbouring an FGFR3 mutation was treated with an inhibitory monoclonal antibody targeting FGFR3. Five PDX were successfully established. Tumor doubling time ranged from 5 to 11 days. Array CGH revealed shared chromosomal aberrations in the patient tumors and PDX. Gene expression microarray and IHC confirmed that PDXs maintain similar patterns to the parental tumors. Tumor growth in the PDX with an FGFR3 mutation was inhibited by the FGFR3 inhibitor. PDXs recapitulate the tumor biology of the patients' primary tumors from which they are derived. Investigations related to tumor biology and drug testing in these models are therefore more likely to be relevant to the disease state in patients. They represent a valuable tool for developing precision therapy in MIBC.

## INTRODUCTION & OBJECTIVES

An estimated 74, 690 incident cases and 15, 580 deaths from bladder cancer are expected to occur in the United States in 2014. This will make it the fourth most common cancer in men and the twelfth most common cancer in women, which is representative also of other industrialized countries [1]. Approximately three quarters of these patients have non-muscle invasive tumors [2], which have a high disposition for recurrence after curative treatment, and a subset is at high risk for progression to invasive disease [3]. The remaining quarter of patients present with muscle invasive bladder cancer (MIBC). Despite optimal surgical therapy, approximately one half of these patients will progress to advanced disease [4]. Response to systemic chemotherapy is rarely durable in these patients and most will succumb to their disease. While targeted therapy has revolutionized the treatment of many cancers [5] no significant breakthroughs have been made for decades to enhance the systemic therapy of MIBC [6].

Analysis of tumor biology by molecular manipulation, identification of relevant diagnostic and predictive biomarkers, and preclinical testing of novel antineoplastic therapeutic agents critically depend on conclusive *in vivo* models of human cancer. For bladder cancer research multiple cell lines are available which reliably grow to tumors after orthotopic inoculation into immunodeficient mice (orthotopic xenograft) [7, 8]. The major shortcoming of these models relates to the cell lines used. During years of cultivation, passaging and expansion, genetic drift has markedly altered these cells from their original genotype and phenotype [9], so that they are no longer truly representative of the disease they are modeling [10]. They likely underrepresent the true tumor heterogeneity seen in patients, which impacts our ability to predict and study therapeutic resistance [11]. Finally xenograft tumors inoculated by injection of cultured cells are deficient of their original stroma which has been shown to highly influence tumor biology and growth [12]. Under these circumstances the ability of current xenografts to predict efficacy of therapeutic agents is limited and *in vivo* observations are only rarely transferrable to the clinical setting.

In order to address these limitations much effort has been made to establish models in which specific genetic characteristics and stromal architecture of the original tumor are reliably retained. These pre-requisites are satisfied by bypassing the establishment of cell lines from human cancers and instead grafting intact cancer tissue into immunocompromised mice (patient derived primary xenografts, PDX). Successful and reliable inoculation of such PDX has, however, proved challenging [13–15]), and satisfactory engraftment rates in most series have only been obtained for highly-aggressive or poorly differentiated cancers [16]. The inoculation of patient tumor under the renal capsule has proven to be most reliable [17], which presumably relates to an abundant blood supply and rapid neo-vascularisation of the grafted tissue. Engraftment rates > 95% have been obtained for several cancer entities with this technique [18, 19], but it has not yet been tested for bladder cancer. The subcutaneous compartment has so far been the most popular graft site for PDX derived from bladder cancer patients, but engraftment rates have been discouraging [16, 20].

In the present study we report our early experience with the establishment of bladder cancer PDX by grafting representative cancer tissue under the renal capsule of immunocompromised mice. Besides demonstrating the feasibility and reproducibility of this model, we have performed extensive molecular characterization of the PDX compared to the patient tumors from which they were derived.

## RESULTS

### Establishment of xenografts from BCA tissue

Patient demographics and clinicopathologic features for the 7 harvested tumors are summarized in Table 1. Successful PDX growth in at least one mouse was observed for all 7 patient-derived tumors implanted under the murine renal capsule. Six PDX grew sufficiently for transfer into further mice. One model (LTL524) had to be excluded after being identified as having human B cell lymphoma originating from transplanted human EBV infected B cells [21]. The 5 transplantable MIBC PDX lines demonstrated doubling times ranging from 5 – 11 days.

**Table 1:** Establishment of transplantable xenograft tumor lines from patient tumors

Patient (age/gender)	Pathologic diagnosis	Pathologic stage/grading	Neodajuvant chemotherapy	Source of tissue graft	Xenograft Model	Establishment of xenograft tumor line	Doubling time of xenograft tumor line
53/male	UC	pT2N0Mx/G2	Gemcitabine/Cisplatin	rCx	LTL392	+	11 days
53/male	UC	pT1N0Mx/G2	-	rCx	LTL480	–	9 days
63/male	UC	pT3bN2Mx/G3	-	rCx	LTL488	+	9 days
72/male	SCC	pT2bN0Mx/G3	-	rCx	LTL489	+	10 days
67/male	UC	pT4bN0Mx/G3	Gemcitabine/Cisplatin	rCx	LTL490	+	4 days
73/male	UC	pT4bN3Mx/G3	Gemcitabine/Carboplatin	rCx	LTL524	+	5 days
70/male	UC	pT4aN3Mx/G3	-	rCx	LTL543	+	9 days

Pathological staging according to the TNM Classification of Malignant Tumours (7th Edition; 2009).

UC = urothelial carcinoma; SCC = squamous cell carcinoma.

### PDXs retain genetic characteristics of patient tumors

#### aCGH

**Figure 1:**
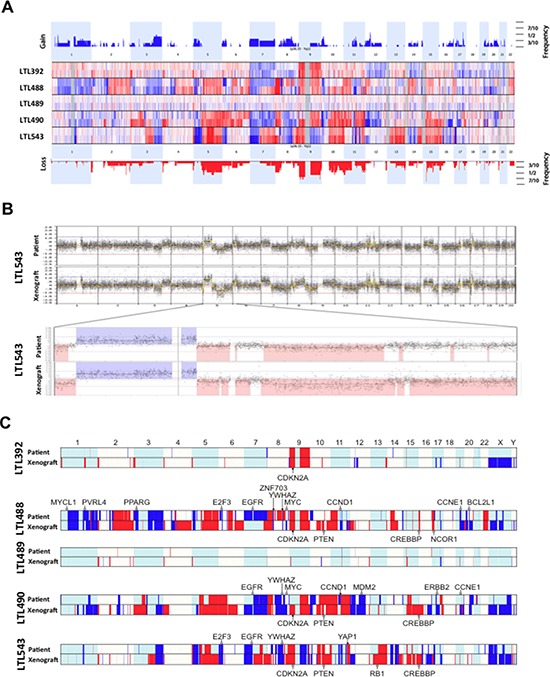
Array comparative genomic hybridisation (aCGH) of primary tumors and corresponding xenografts **A.** In this depiction, the somatic chromosomes are lined up in numeric order from left to right. Each horizontal line separates one pair (patient tumor above, PDX below) from the next. Any elevation above the baseline represents a copy number gain (blue), and any depression below the baseline represents a loss (red). Slight differences in some genomic areas are explained by the loss of cell heterogeneity (decontamination from macrophages and leucocytes) in the PDX tumors. A superkaryogram of all 5 primary tumors and corresponding PDX is illustrated at the top and bottom of this figure (copy number gain as blue bars, loss as red bars). The thickness of the bars corresponds to the quantity of pairs making up the defect. **B.** Detailed illustration of the whole genome of patient and corresponding PDX of model AB543 with magnification of chromosome 5. The similarity is representative of all pairs. **C.** Genome copy number calls for the five matched patient-xenograft pairs showing genes frequently altered in bladder cancer. Genes that fall within regions of significant GISTIC peaks in the TCGA study [22] are annotated, with open circles demonstrating single copy gain/loss, and filled circles highlighting genes that are amplified / homozygously lost. Note that some regions of copy number variance can only be robustly called in the xenografts. This is likely to reflect the high purity of these tumors relative to the patient tumors, where normal admixture and heterogeneity can dampen copy number signals.

We performed aCGH on all samples and revealed striking concordance in the copy number profiles between patient tumors and their matched PDX [Figure 1A, 1B]. Copy number changes in the PDX models were more prominent, presumably due to reduction in the contribution of non-tumor cells such as macrophages and lymphocytes. The breakpoints within the resolution of the technology were located in identical locations. In the recent Cancer Genome Atlas (TCGA) study of bladder urothelial carcinoma [22], 20 genes were flagged as falling within statistically significant focal copy number peaks across the 131 tumors examined. Remarkably, 19 of these 20 genes showed congruent copy number change in at least one of our xenograft tumors, although only four genes were affected by high copy gain or homozygous loss (CDKN2A, CCND1, ZNF703 and YAP1) [Figure 1C]. Especially the copy number loss of CDKN2A in all models histologically classified as urothelial carcinoma (4/5 models) is in concordance with the TCGA data where 47% of non-squamous tumors exhibit this specific alteration. This demonstrates the potential utility of our models to recapitulate the chromosomal copy number variation commonly detected in patient tumors.

#### Mutation analysis FGFR3 gene

As mutations in the FGFR3 regularly occur in urothelial cancer of the bladder and are amenable to targeted therapies the primary tumors and their corresponding PDX were analyzed for mutations in this gene by direct sequencing. Only one patient tumor (LTL392) harbored a mutation, which was located in exon 7 (S249C). This was retained in the corresponding PDX [Figure 4A].

### PDXs retain transcriptional and morphologic characteristics of patient tumors

#### Gene expression profiling

**Figure 2:**
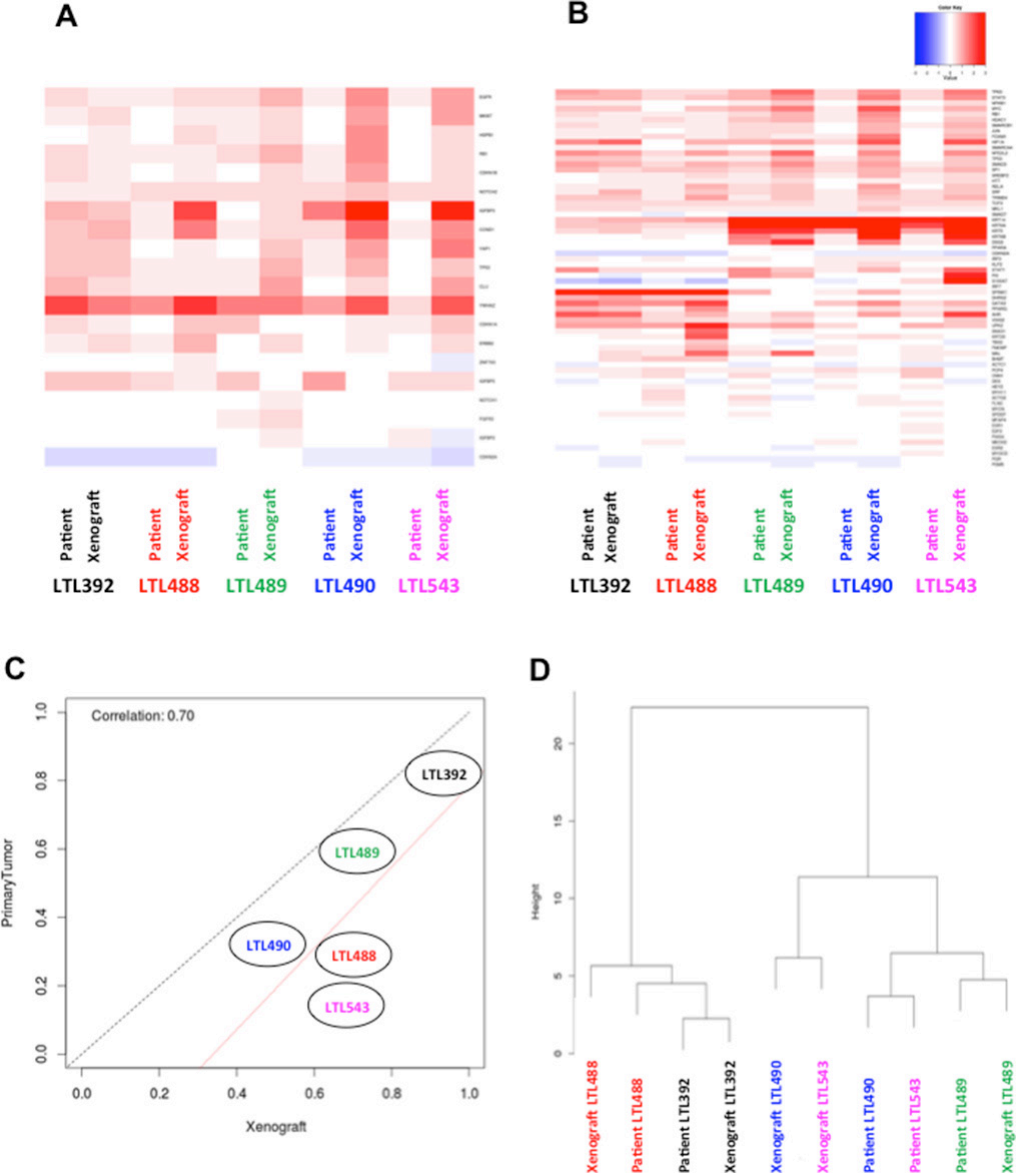
Retention of genetic expression patterns in xenografts Heat maps of gene expression from potential target substrates **A.** and bladder cancer related genes **B.** show no single gene that is highly differentially expressed between patient tumor and matched PDX. **C.** The RF15 scores for patient tumor samples versus their corresponding PDX illustrate a high correlation in 3 of 5 cases, and a trend towards more aggressive phenotype in 2 PDX. The red line is the line of best fit, and the black line indicates perfect correlation. **D.** A cluster dendogram was designed using 118 genes related to bladder cancer. Sample pairs (primary tumors and corresponding PDX) tended to cluster together, except for LTL490 and LTL543. The matched PDX - primary tumor pairs are represented with the labels of the same color.

Analysis of gene expression was performed in patient tumors and corresponding PDX for 17 common genes whose substrates are potentially amenable for targeted therapy [Figure 2A] and for a selection of 68 bladder cancer related genes associated with distinct subtypes of invasive bladder cancer [23] [Figure 2B]. The core level gene expression was used to build heatmaps that show no single gene is highly differentially expressed between patient tumor and matched PDX suggesting that patient tumor and paired PDX have similar genomic profiles of bladder genes. The RF15 scores for primary tumor samples versus their corresponding PDX illustrate a high correlation, although the PDX demonstrated overall a higher risk than the patient tumor [Figure 2C]. This high correlation between the scores suggests that the genomic profile of the primary tumor is preserved in the PDX; however the shift towards higher scores in the PDX samples might be a reflection of less stromal tissue and a more homogonous cellular population found in the PDX. A clustering of the samples was conducted based on the expression of 118 bladder cancer genes [Figure 2D]. This gene set created by integrating three sources: (1) the 17 genes representing potential targets for therapy, (2) the 68 genes associated to distinct subgroups of invasive bladder cancer, and (3) 42 bladder cancer genes from KEGG (Kyoto Encyclopedia of Genes and Genomes) pathways. This clustering revealed that corresponding pairs (patient tumors and corresponding PDX) tended to cluster together, although primary tumors LTL490 and LTL543 as well as PDX LTL490 and LTL543 clustered together. Differential expression analysis conducted at the probeset level using Fold Change method revealed that some patients have distinct transcriptome profiles compared to their paired PDX. For example, more than 2000 probesets (out of 1.4M) are differentially expressed (Fold change = 2) between LTL543 and LTL490 and their paired PDX. LTL489, LTL392 and LTL488, on the other hand, have less than 200 probesets differentially expressed in comparison to the paired PDX. Of the 2266 probesets differentially expressed in LTL543, the vast majority (2194) were overexpressed in the PDX model compared to the primary tumor. Interestingly, MALAT and NEAT1 were among the most overexpressed probesets in the PDX model, and IGKC was among the most downregulated genes. IGKC was found also to be downregulated in the other four PDXs. These changes are consistent with a selection of more aggressive tumor features in the PDX compared to the primary tumor.

#### Histology and immunohistology

**Figure 3:**
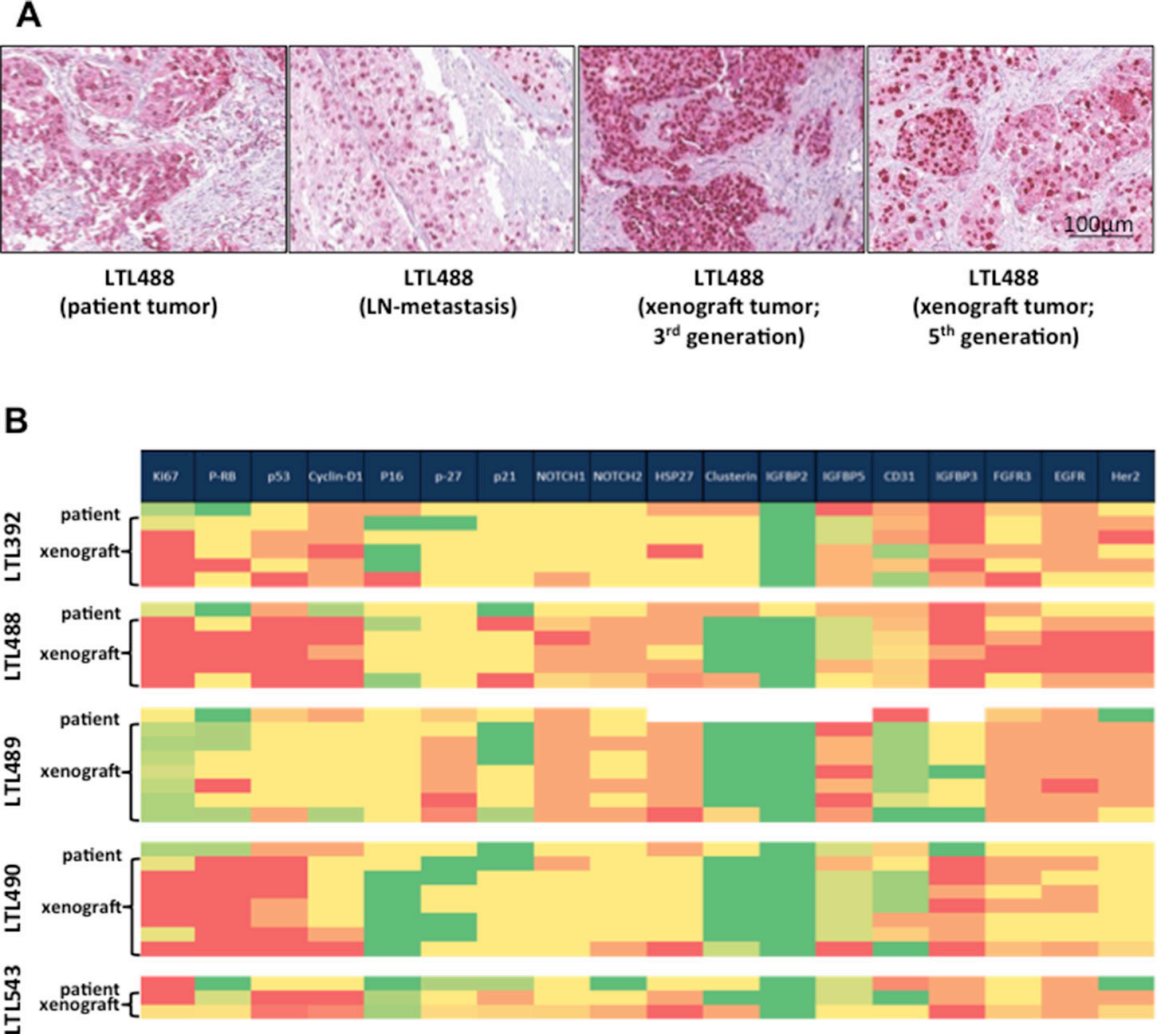
Retention of morphological characteristics and protein expression in xenografts **A.** Representative areas of each patient tumor, corresponding patient lymph node metastasis if present and different generations of the corresponding PDX were stained with H&E. Here patient and the corresponding xenograft tumor of model LTL488 are shown as an example. **B.** Results of immunohistochemical staining for several potential targets and other tumor biomarkers depicted on a heat map. Higher protein expression is illustrated in red, and lower expression in green. Expression levels of most analyzed proteins (except p-RB, Her-2 and CD31) in primary tumor were retained by different generations of the corresponding PDX.

Microscopic examination of patient tumors and corresponding PDX of different generations revealed retention of morphological characteristics (cell and tissue architecture). H&E sections of a patient tumor (LTL488), its corresponding lymph node metastasis and PDX are exemplarily depicted in Figure 3A. The analysis of immunohistological features was compiled in a heatmap [Figure 3B]. Staining intensity showed a close correlation for various proteins (except p-RB, Her-2 and CD31) between patient tumors and their corresponding PDX.

### A model for evidence-based precision oncology

**Figure 4:**
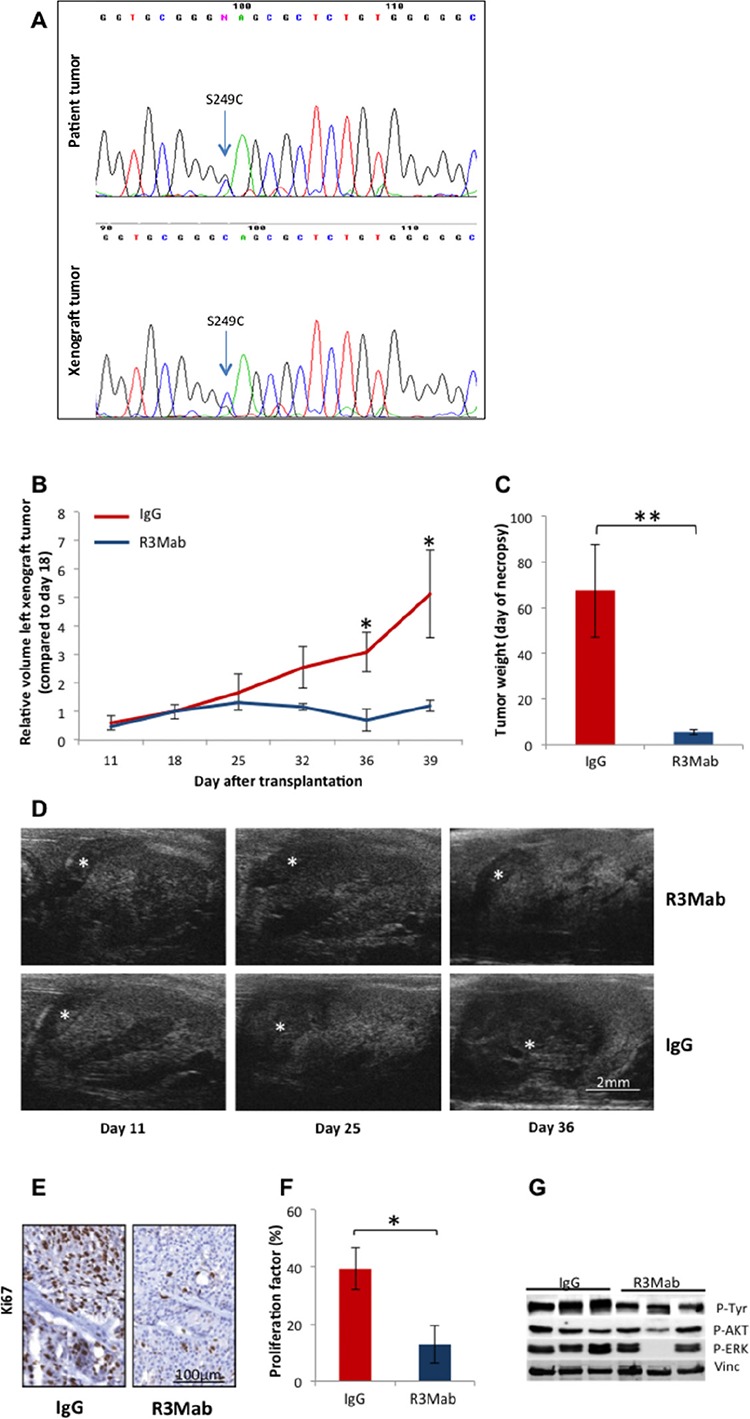
Precision therapy with R3Mab in model LTL392 **A.** Sequencing for mutations in FGFR3 gene revealed a mutation in exon 7 in patient and corresponding PDX of model LTL392, with the codon shift from TCC to TGC causing the replacement of serine by cysteine in amino acid position 249 of the protein. **B.** Targeted therapy with the monoclonal antibody R3Mab compared to control (IgG) was initiated at day 18 after PDX-transplantation under the renal capsule. Measurements of tumor volumes by ultrasonography **D.** demonstrated significantly inhibited tumor growth (*) in the treatment group from day 36 forward (*p* < 0.05). **C.** Tumor weight at the end of the experiment (day 41) significantly differed between the groups (*p* < 0.01). **E.**, **F.** Representative PDX tissue was stained for the proliferative marker Ki67. The proliferation index stated in percentage of stained nulei for Ki67 was significantly higher in the control group (IgG) compared to the treatment group (R3Mab). **G.** Protein-analysis of PDX tissue by Western blot showed a reduction of phosphorylated tyrosine and downstream substrates of FGFR3 signalling after treatment with inhibiting antibody.

*In vivo* treatment of PDX model LTL392 with an inhibiting antibody targeting FGFR3 (R3Mab) strongly inhibited tumor growth. Significantly smaller tumor volumes were observed after 18 days of treatment with R3Mab compared to control antibody, and these differences persisted until the end of the experiment (*p* < 0.05 [Figure 4B, 4D]). Accordingly, tumor weight at necropsy differed significantly between the two groups (*p* < 0.01 [Figure 4C]). Evaluation of the harvested PDX samples by immunohistochemistry revealed a significant decrease in the proliferation index in the treatment group compared to control (*p* < 0.05 [Figure 4E, 4F). Western blotting showed inhibition of pTyr and FGFR3 downstream signaling, including p-Akt and p-Erk1/2, after targeting FGFR3 with R3Mab.

## DISCUSSION

The existence of animal models that reliably mimic human malignancies is a basic requirement in oncologic research for the identification of molecular targets and subsequent development of novel targeted therapies [24]. *In vivo* models of individual patient-derived cancers represent the gold standard in order to personalize therapy by determining chemosensitivity prior to clinical administration of a therapeutic agent [25]. In this study we have successfully established PDX from patients with bladder cancer, which retained genetic and morphological characteristics of the original tumor. We have demonstrated that these are suitable tools for drug efficacy studies.

Tissue grafts of several different tumor types including bladder cancer have previously been transplanted into various different compartments and organ sites in mice [13–15, 26]. In most cases, however, successful passage rates were low and the majority of established transplantable PDX lines originated from less differentiated or anaplastic primary tumors. This is true also for bladder cancer, which has previously been grafted primarily in the subcutaneous compartment [26], and successful passage rates even in the most recent studies have only been acceptable in very aggressive cancers [16, 20].

Our approach to inoculate and transplant representative tumor tissue under the renal capsule builds on success establishing similar PDX from tumors derived from other organs [17–19], and addresses most limitations of existing models. We were able to establish transplantable PDX lines in 71.4%, with one of the two failures being due to concomitant establishment of lymphoma in the host mouse. This constitutes a remarkable increase compared to previous models [16]. Although the subcapsular renal site is not the physiologic, orthotopic location for bladder cancer, it has emerged as the optimal environment for PDX survival and growth. We have not attempted primary implantation in the bladder due to the presumed technical challenge. We recognize that our PDX are derived from a selection of high risk tumors (disproportionately high representation of pT4 and pN+ tumors), which was not intentional. Nevertheless we were able to demonstrate that even our few models recapitulated a large proportion of the chromosomal copy variation commonly detected in bladder cancer [22]. We are currently developing PDX from lower risk tumors, including non-muscle invasive tumors.

As PDX lines are established by the grafting of representative tumor pieces that contain stromal cells and extracellular matrix in addition to cancerous epithelium, mutual interactions between these components maintain tumor biology representative of the original patient tumor [12]. Histological analyses clearly demonstrated in our models that specific histological features such as tissue architecture and cellular morphology are maintained for up to 13 generations of propagation. The same is true at a gene copy number (aCGH), RNA expression (microarray) and protein expression (IHC) level. This implies that drug efficacy or other molecular studies in this model are more likely to correlate to similar studies in patients. One particularly attractive feature of this model is the ability to grow patient tumor in a mouse and test the efficacy of specific candidate drugs before administering these drugs to the patient. While such modeling does take time and patients usually require immediate therapy, this paradigm would be feasible in patients with residual MIBC in their cystectomy specimen after neoadjuvant chemotherapy. These patients have a high risk of subsequent systemic failure, but usually receive no immediate adjuvant therapy due to a lack of efficacious agents. They have abundant tumor tissue available for molecular analysis and primary xenografting. Drugs can be tested in the PDX model and subsequently administered to the patient at the time of recurrence. This remains a concept that requires rigorous testing.

A principal limitation of current PDX models of bladder cancer is their inability to metastasize. Our experience with orthotopic injection of human bladder cancer cell lines has demonstrated that local growth in the bladder causes morbidity requiring euthanasia prior to metastasis [8]. While we did not observe metastasis in any of our bladder cancer PDX, we have done so in other tumor systems, indicating that this model is suitable for the study of metastasis, and we continue to establish bladder cancer PDX with the intent of developing metastatic lines.

The principal limitation of our study is the small number of established PDX lines and the fact that a drug efficacy study was performed in only one of these models, and we have not yet taken the step of testing a drug in this model prior to administration in a patient with bladder cancer. We have, however, extended our model system for PDX under the renal capsule to include bladder cancer, and have demonstrated that these models closely resemble the original patient tumor from which they were derived.

## CONCLUSIONS

We have successfully developed patient-derived xenografts of bladder cancer which reliably retain specific genetic and morphological features of the primary patient tumors. The ability to identify potential molecular targets and to test the efficacy of targeting agents in this model has been demonstrated. We speculate that this will be a valuable tool for developing molecularly targeted precision therapy in patients with bladder cancer in the future.

## MATERIALS AND METHODS

### Patient tumor samples

Between 2010 and 2012 tissue specimens from 7 patients undergoing radical cystectomy for MIBC at Vancouver General Hospital were obtained. The study was approval by the Clinical Research Ethics Board (Protocol number: A10-0350) and each patient provided informed consent for use of his/her tissue. Representative tumor tissue was immediately cut into small pieces for further processing after harvesting from the patient.

### Animals and tumor transplantations

All animal procedures were performed according to the guidelines of the Canadian Council on Animal Care (CCAC). The protocol was approved by the Animal Care Committee of the University of British Columbia (Protocol Number: A10-0350). Fresh human tumor pieces were bilaterally grafted under the renal capsule of female NOD-SCID mice and passaged serially for establishment of bladder cancer PDX as previously described [19]. PDX tumors were harvested at humane endpoints and processed for further analysis.

### Histology and tissue microarray construction

Pieces of patient and PDX tissue were fixed in formalin and embedded in paraffin. 4 μm thick sections were stained with haematoxylin and eosin (H&E) and analysed by a genitourinary pathologist (L.F.) with respect to tumor morphology. A tissue microarray (TMA) was constructed of 10mm duplicate cores originating from representative areas of patient and PDX tumors. For immunohistochemistry the following primary antibodies were applied to 4 μm sections: mouse monoclonal anti-CD31 (1:50; M0823, DAKO (Burlington, Ontario, Canada)), goat polyclonal anti-Clusterin-α (1:1000, sc-6420, Santa Cruz (Dallas, TX)), rabbit monoclonal anti-cyclin D1 (1:100, RM-9104, Thermo Scientific (Rockford, IL)), rabbit monoclonal anti-EGFR (1:25, #4267, Cell Signaling (Danvers, MA)), rabbit polyclonal anti-FGFR3 (1:600, F0425, Sigma-Aldrich (Oakville, ON, Canada)), rabbit monoclonal anti-Her2 (1:25, #2165, Cell Signaling), rabbit monoclonal anti-HSP27 (1:3000, SPA-803, Enzo Life Sciences (Farmingdale, NY)), mouse monoclonal anti IFGBP2 (1:100, sc-365368, Santa Cruz), mouse monoclonal anti-IGFBP3 (1:1000, sc-365936, Santa Cruz), rabbit polyclonal anti-IGFBP5 (1:25, sc-13093, Santa Cruz), rabbit monoclonal anti-Ki67 (1:500; RM-9106, Thermo Scientific), rabbit monoclonal anti-Notch1 (1:25, #3608, Cell Signaling), rabbit monoclonal anti-Notch2 (1:100, #5732, Cell Signaling), rabbit polyclonal anti-phospho-Rb (1:200, #9308, Cell Signaling), rabbit polyclonal anti-P16 (1:1000, #10883-1-AP, Proteintech (Chicago, IL)), rabbit polyclonal anti-P21 (1:150, sc-397, Santa Cruz), rabbit polyclonal anti-P27(1:50, sc-528, Santa Cruz)), and mouse monoclonal anti-P53 (1:3000, M7001, DAKO (Burlington, ON, Canada)). Staining was performed with a corresponding secondary antibody by the Ventana autostainer model Discover XT (Ventana Medical System (Tuscon, AZ, USA)) which included an enzyme-labeled biotin streptavidin system and solvent-resistant 3, 30-diaminobenyidine Map kit. Specific protein expression was detected at 20x magnification and graded on a four point scale (0–3). For assessment of proliferative index Ki67 was analysed at 40x magnification in a minimum of five randomly selected high-power fields and staining intensity estimated in percentage (number of positive stained nuclei for Ki67 to total number of nuclei in tumor). Additionally tumor neovascularisation was determined by staining for CD31 (average count of blood vessels in five high-power fields).

### Protein extraction and western blot analysis

Samples containing 40mg of protein from lysates of harvested PDX were separated by SDS-PAGE on 10% Tris-HCl gels and subsequently transferred to nitrocellulose filters. After blocking (Odyssey Blocking buffer; LI-COR Biosciences (Lincoln, NE)) the blots were incubated with the following primary antibodies: rabbit monoclonal anti-phospho-Akt (1:1000, #4060, Cell Signalling), mouse monoclonal anti- phospho- Erk1/2 (1:1000, #4374, Cell Signalling), mouse monoclonal anti-phosphotyrosine (1:1000, 05-321X, EMD Milipore (Darmstadt, Germany) and rabbit polyclonal anti-Vinculin (1:2, 500, PA5-19842, Thermo Scientific). Subsequently the filters were washed with phosphate-buffered saline (PBS) containing 0.1% Tween and then incubated with Alexa Fluor secondary antibodies (1:5, 000; Invitrogen). Specific proteins were detected using Odyssey IR imaging system (LI-COR Biosciences).

### RNA/DNA isolation

Fresh patient tumor and PDX specimens were immediately frozen after harvesting and stored at −80°C. After thawing, RNA and genomic DNA was isolated in a clean environment by the RNeasy and DNeasy^®^ Tissue Kit (QIAGEN (Valencia, CA)), respectively. RNA and DNA purity was validated by measuring the ratio of 260nm/280nm absorbance with a Nanodrop 2000 spectrophotometer (Thermo Scientific). Samples with a ratio under 1.87 were excluded from further analysis.

### Gene expression analysis

RNA was amplified and labeled using the Ovation WTA FFPE system (NuGen (San Carlos, CA)) and hybridized to GeneChip Human Exon 1.0 ST oligonucleotide microarrays (Affymetrix (Santa Clara, CA)) according to the manufacturer's recommendations. Microarray quality control was performed using Affymetrix power tools and custom metrics. Normalization and core level summarization of the microarray data was performed using SCAN [27]. The generated data can be accessed at the GEO (ID: GSE67312). Hierarchical clustering, using Euclidean distance as a dissimilarity metric and Ward function as agglomeration method, is used to assess if the matching PDX - primary tumor sample pairs have a similar genomic profile. A previously developed 15-marker genomic signature (RF15 score) predictive of MIBC recurrence was used to determine if the genomic risk score would be robust in the PDX samples. This signature was developed using a cohort of 133 patients with organ-confined disease who underwent radical cystectomy between 1998 and 2004 [23]. In a subsequent validation cohort of 66 patients the signature achieved an area under the receiver operating characteristic curve (AUC) of 0.77 [95% CI: 0.65–0.91] [28]. This model was used as a genomic test to make a comparison between risk of recurrence associated with the gene expression found in the PDX samples and primary tumor samples.

### FGFR3 mutation analysis

Exons 7, 10 and 15 were amplified by PCR using Platinum Pfx DNA Polymerase (Life Technologies (Burlington, ON, Canada)). The following primers were used: 5′-CGGCAGTGGCGGTGGTGGTG-3′ (sense) and 5′-AGCACCGCCGTCTGGTTGGC-3′ (antisense) for exon 7, 5′-CCTCAACGCCCATGTCTTT-3′ (sense) and 5′-AGGCAGCTCAGAACCTGGTA-3′ (antisense) for exon 10 and 5′- GATGATCGGGAAACACAAA-3′ (sense) and 5′-TAGACTCGGTCAAACAAGG-3′ (antisense) for exon 15 (Sigma-Aldrich (St. Louis, MO)). Cycling variables were set as following: 5min at 94°C, 35 cycles of 15sec at 94°C, 30sec at 68°C, followed by 5 min at 68°C and then a hold at 4°C (exon 7); 5min at 94°C, 35 cycles of 15sec at 94°C, 30sec at 60°C, 30sec at 68°C, followed by 5 min at 68°C and then hold at 4°C (exon 10 and 15) [29]. PCR products were purified using Qiagen MinElute spin columns, quantified by Nanodrop, and then sequenced directly with 400nM 7R primer (exon 7), 10R primer (exon 10) or 15F primer (exon 15) by BigDye Terminator Cycle Sequencing (Life Technologies) on an ABI 310 Genetic Analyzer (Applied Biosystems (Burlington, ON, Canada)). Cycle sequencing conditions were set as following: 96°C 1min, 25 cycles of 96°C 15sec, 62°C 30sec, 65°C for 2.5 min (exon 7); 96°C 1 min, 25 cycles of 96°C 15 sec, 55°C 30sec, 65°C 3min (exon 10 and 15). Sequence chromatograms were analyzed manually for the presence/absence of the SNP at known locations. S249C SNP in Exon 7 was confirmed by sequencing with 7R primer by NAPS (Nucleic Acid Processing Service) at Michael Smith Laboratories at UBC (Vancouver, BC, Canada).

### Array comparative genomic hybridization

Array comparative genomic hybridization (aCGH) was performed on the Agilent Human Genome CGH Microarray^®^ platform (Agilent (Santa Clara, CA)) at the VPC. Genomic DNA from each patient tumor and corresponding PDX was quantified by the Nanodrop 2000^®^ spectrophotometer (ThermoScientific) and a quantity of 0.5 μg was fluorescently labeled according to the NimbleGen enzymatic labeling protocol (NimbleGen Arrays User Guide CGH Analysis v6.0, Roche NimbleGen (Madison, WI)). 5 μg of each Cy5-labeled sample was co-hybridized with 5 μg of gender matched Cy3-labeled human reference DNA (Promega (Madison, WI)) on Agilent SurePrint G3 Human^®^ CGH 8×60K microarrays (AMDID 021924). Arrays were scanned with the Agilent DNA Microarray Scanner at a 3 μm scan resolution, and quantified with Feature Extraction^®^ 10.0.1.1 software (Agilent). CGH processed signal was then uploaded into Nexus CGH^®^ software (Biodiscovery (Hawthorne, CA)) and processed using BioDiscovery's FASST2 Segmentation Algorithm to estimate copy number state. These state values were then used to make calls based on a log-ratio threshold. The significance threshold for segmentation was set at 5.0E-6 also requiring a minimum of 3 probes per segment and a maximum probe spacing of 1000 between adjacent probes before breaking a segment. The log ratio thresholds for single copy gain and single copy loss were set at 0.2 and −0.23, respectively. The log ratio thresholds for two or more copy gain and homozygous loss were set at 1.14 and −1.1 respectively. Upon loading of raw data files, signal intensities are normalized via division by mean. All samples are corrected for genomic control (GC) wave content using a systematic correction algorithm. All samples were additionally analyzed specifically for copy number changes of genes that fall within regions of significant GISTIC (genomic identification of significant targets in cancer) peaks identified in a recent report from the Cancer Genome Atlas (TCGA) [22].

### *In vivo* monitoring of xenograft growth

Measurement of tumor growth was performed with the Vevo 770^®^ small animal imaging platform (Visual Sonics (Toronto, ON, Canada)). A high frequency RMV 706 ultrasound scanhead (20 – 60 MHz), which allowed a lateral resolution of 30 micron and frame rates up to 240 fps, was used. 3D ultrasound was performed with scanning of the tumor as a whole in 0.1mm steps. The tumor volume was determined using the Visual Sonics imaging software package by analysis of every fifth picture according to the user manual [30].

### *In vivo* efficacy study of targeted therapy

FGFR3 targeting antibody R3Mab [31] and isotype control antibody (human IgG1) were provided by Genentech (South San Francisco, CA). For study of drug efficacy, the PDX line LTL392B (4^th^ generation) was transplanted into 10 animals. On day 18, after reaching an average tumor volume of 115.3mm^3^, the animals were randomized into 2 treatment groups based on tumor burden. R3Mab (30mg/kg) or control antibody (30mg/kg) was injected intraperitoneally twice weekly for 3 weeks. Quantification of tumor response was performed by ultrasound imaging (1x/week) and determination of tumor burden at necropsy on day 41 [19] For statistical analysis the mean tumor weights with their standard deviations were calculated and the significance of differences measured by Student's *t* test (GraphPad Software Inc. (San Diego, CA). *P* < 0.05 was considered significant.

## List of abbreviations

CN: Copy number
CNV: Copy number variation
GC: Genomic control
GISTIC: Genomic identification of significant targets in cancer
H&E: Hematoxylin and eosin
MIBC: Muscle invasive bladder cancer
PBS: Phosphate-buffered saline
PCR: Polymerase chain reaction
rCx: Radical cystectomy
SCC: Squamous cell carcinoma
TCGA: The Cancer Genome Atlas Research Network
TURBT: Transurethral resection bladder tumor
UC: Urothelial carcinoma

## References

[1] Siegel R, Ma J, Zou Z, Jemal A (2014). Cancer statistics. CA Cancer J Clin.

[2] Babjuk M, Burger M, Zigeuner R, Shariat SF, van Rhijn BW, Comperat E, Sylvester RJ, Kaasinen E, Bohle A, Palou Redorta J, Roupret M (2013). EAU guidelines on non-muscle-invasive urothelial carcinoma of the bladder: update 2013. Eur Urol.

[3] Sylvester RJ, van der Meijden AP, Oosterlinck W, Witjes JA, Bouffioux C, Denis L, Newling DW, Kurth K (2006). Predicting recurrence and progression in individual patients with stage Ta T1 bladder cancer using EORTC risk tables: a combined analysis of 2596 patients from seven EORTC trials. Eur Urol.

[4] Shariat SF, Karakiewicz PI, Palapattu GS, Lotan Y, Rogers CG, Amiel GE, Vazina A, Gupta A, Bastian PJ, Sagalowsky AI, Schoenberg MP, Lerner SP (2006). Outcomes of radical cystectomy for transitional cell carcinoma of the bladder: a contemporary series from the Bladder Cancer Research Consortium. J Urol.

[5] Slamon D, Eiermann W, Robert N, Pienkowski T, Martin M, Press M, Mackey J, Glaspy J, Chan A, Pawlicki M, Pinter T, Valero V, Liu MC, Sauter G, von Minckwitz G, Visco F (2011). Adjuvant trastuzumab in HER2-positive breast cancer. N Engl J Med.

[6] von der Maase H, Sengelov L, Roberts JT, Ricci S, Dogliotti L, Oliver T, Moore MJ, Zimmermann A, Arning M (2005). Long-term survival results of a randomized trial comparing gemcitabine plus cisplatin, with methotrexate, vinblastine, doxorubicin, plus cisplatin in patients with bladder cancer. J Clin Oncol.

[7] Chan E, Patel A, Heston W, Larchian W (2009). Mouse orthotopic models for bladder cancer research. BJU Int.

[8] Jager W, Moskalev I, Janssen C, Hayashi T, Awrey S, Gust KM, So AI, Zhang K, Fazli L, Li E, Thuroff JW, Lange D, Black PC (2013). Ultrasound-guided intramural inoculation of orthotopic bladder cancer xenografts: a novel high-precision approach. PLoS One.

[9] Masramon L, Vendrell E, Tarafa G, Capella G, Miro R, Ribas M, Peinado MA (2006). Genetic instability and divergence of clonal populations in colon cancer cells *in vitro*. J Cell Sci.

[10] Sausville EA, Burger AM (2006). Contributions of human tumor xenografts to anticancer drug development. Cancer Res.

[11] Guey LT, Garcia-Closas M, Murta-Nascimento C, Lloreta J, Palencia L, Kogevinas M, Rothman N, Vellalta G, Calle ML, Marenne G, Tardon A, Carrato A, Garcia-Closas R, Serra C, Silverman DT, Chanock S (2010). Genetic susceptibility to distinct bladder cancer subphenotypes. Eur Urol.

[12] Bhowmick NA, Neilson EG, Moses HL (2004). Stromal fibroblasts in cancer initiation and progression. Nature.

[13] Verschraegen CF, Hu W, Du Y, Mendoza J, Early J, Deavers M, Freedman RS, Bast RC, Kudelka AP, Kavanagh JJ, Giovanella BC (2003). Establishment and characterization of cancer cell cultures and xenografts derived from primary or metastatic Mullerian cancers. Clin Cancer Res.

[14] Schumacher U, Adam E, Horny HP, Dietl J (1996). Transplantation of a human ovarian cystadenocarcinoma into severe combined immunodeficient (SCID) mice—formation of metastases without significant alteration of the tumour cell phenotype. Int J Exp Pathol.

[15] Kiguchi K, Kubota T, Aoki D, Udagawa Y, Yamanouchi S, Saga M, Amemiya A, Sun FX, Nozawa S, Moossa AR, Hoffman RM (1998). A patient-like orthotopic implantation nude mouse model of highly metastatic human ovarian cancer. Clin Exp Metastasis.

[16] Park B, Jeong BC, Choi YL, Kwon GY, Lim JE, Seo SI, Jeon SS, Lee HM, Choi HY, Lee KS (2013). Development and characterization of a bladder cancer xenograft model using patient-derived tumor tissue. Cancer Sci.

[17] Bogden AE, Haskell PM, LePage DJ, Kelton DE, Cobb WR, Esber HJ (1979). Growth of human tumor xenografts implanted under the renal capsule of normal immunocompetent mice. Exp Cell Biol.

[18] Cutz JC, Guan J, Bayani J, Yoshimoto M, Xue H, Sutcliffe M, English J, Flint J, LeRiche J, Yee J, Squire JA, Gout PW, Lam S, Wang YZ (2006). Establishment in severe combined immunodeficiency mice of subrenal capsule xenografts and transplantable tumor lines from a variety of primary human lung cancers: potential models for studying tumor progression-related changes. Clin Cancer Res.

[19] Lee CH, Xue H, Sutcliffe M, Gout PW, Huntsman DG, Miller DM, Gilks CB, Wang YZ (2005). Establishment of subrenal capsule xenografts of primary human ovarian tumors in SCID mice: potential models. Gynecol Oncol.

[20] Hofner T, Macher-Goeppinger S, Klein C, Rigo-Watermeier T, Eisen C, Pahernik S, Hohenfellner M, Trumpp A, Sprick MR (2013). Development and characteristics of preclinical experimental models for the research of rare neuroendocrine bladder cancer. J Urol.

[21] Chen K, Ahmed S, Adeyi O, Dick JE, Ghanekar A (2012). Human solid tumor xenografts in immunodeficient mice are vulnerable to lymphomagenesis associated with Epstein-Barr virus. PLoS One.

[22] Cancer Genome Atlas Research N (2014). Comprehensive molecular characterization of urothelial bladder carcinoma. Nature.

[23] Choi W, Porten S, Kim S, Willis D, Plimack ER, Hoffman-Censits J, Roth B, Cheng T, Tran M, Lee IL, Melquist J, Bondaruk J, Majewski T, Zhang S, Pretzsch S, Baggerly K (2014). Identification of distinct basal and luminal subtypes of muscle-invasive bladder cancer with different sensitivities to frontline chemotherapy. Cancer Cell.

[24] Voskoglou-Nomikos T, Pater JL, Seymour L (2003). Clinical predictive value of the *in vitro* cell line, human xenograft, and mouse allograft preclinical cancer models. Clin Cancer Res.

[25] Dong X, Guan J, English JC, Flint J, Yee J, Evans K, Murray N, Macaulay C, Ng RT, Gout PW, Lam WL, Laskin J, Ling V, Lam S, Wang Y (2010). Patient-derived first generation xenografts of non-small cell lung cancers: promising tools for predicting drug responses for personalized chemotherapy. Clin Cancer Res.

[26] Sufrin G, McGarry MP, Sandberg AA, Murphy GP (1979). Heterotransplantation of human transitional cell carcinoma in athymic mice. J Urol.

[27] Piccolo SR, Sun Y, Campbell JD, Lenburg ME, Bild AH, Johnson WE (2012). A single-sample microarray normalization method to facilitate personalized-medicine workflows. Genomics.

[28] Mitra AP, Lam LL, Ghadessi M, Erho N, Vergara IA, Alshalalfa M, Buerki C, Haddad Z, Sierocinski T, Triche TJ, Skinner EC, Davicioni E, Daneshmand S, Black PC (2014). Discovery and validation of novel expression signature for postcystectomy recurrence in high-risk bladder cancer. J Natl Cancer Inst.

[29] Jebar AH, Hurst CD, Tomlinson DC, Johnston C, Taylor CF, Knowles MA (2005). FGFR3 and Ras gene mutations are mutually exclusive genetic events in urothelial cell carcinoma. Oncogene.

[30] Olson P, Chu GC, Perry SR, Nolan-Stevaux O, Hanahan D (2011). Imaging guided trials of the angiogenesis inhibitor sunitinib in mouse models predict efficacy in pancreatic neuroendocrine but not ductal carcinoma. Proc Natl Acad Sci U S A.

[31] Qing J, Du X, Chen Y, Chan P, Li H, Wu P, Marsters S, Stawicki S, Tien J, Totpal K, Ross S, Stinson S, Dornan D, French D, Wang QR, Stephan JP (2009). Antibody-based targeting of FGFR3 in bladder carcinoma and t(4, 14)-positive multiple myeloma in mice. J Clin Invest.

